# Addition of zirconium oxide to Biodentine increases radiopacity and does not alter its physicochemical and biological properties

**DOI:** 10.1590/1678-7757-2018-0429

**Published:** 2019-03-27

**Authors:** Victor Manuel Ochoa-Rodríguez, Mario Tanomaru-Filho, Elisandra Marcia Rodrigues, Juliane Maria Guerreiro-Tanomaru, Rubens Spin-Neto, Gisele Faria

**Affiliations:** 1 Universidade Estadual Paulista - UNESP, Faculdade de Odontologia de Araraquara, Departamento de Odontologia Restauradora, Araraquara, São Paulo, Brasil.; 2 University of Aarhus, Department of Dentistry, Oral Radiology, Aarhus, Denmark.

**Keywords:** Dental cements, Dental digital radiography, Endodontics, Materials testing, Physicochemical analysis

## Abstract

**Objectives:**

To evaluate the radiopacity of Biodentine (BD) and BD associated with 15% calcium tungstate (BDCaWO_4_) or zirconium oxide (BDZrO_2_), by using conventional and digital radiography systems, and their physicochemical and biological properties.

**Materials and Methods:**

Radiopacity was evaluated by taking radiographs of cement specimens (n=8) using occlusal film, photostimulable phosphor plates or digital sensors. Solubility, setting time, pH, cytocompatibility and osteogenic potential were also evaluated. Data were analyzed using one-way ANOVA and Tukey post-test or two-way ANOVA and Bonferroni post-test (α=0.05).

**Results:**

BD radiopacity was lower than 3 mm Al, while BD ZrO_2_ and BD CaWO_4_ radiopacity was higher than 3 mm Al in all radiography systems. The cements showed low solubility, except for BDCaWO_4_. All cements showed alkaline pH and setting time lower than 34 minutes. MTT and NR assays revealed that cements had greater or similar cytocompatibility in comparison with control. The ALP activity in all groups was similar or greater than the control. All cements induced greater production of mineralized nodules than control.

**Conclusions:**

Addition of 15% ZrO_2_ or CaWO_4_ was sufficient to increase the radiopacity of BD to values higher than 3 mm Al. BD associated with radiopacifiers showed suitable properties of setting time, pH and solubility, except for BDCaWO_4_, which showed the highest solubility. All cements had cytocompatibility and potential to induce mineralization in Saos-2 cells. The results showed that adding 15% ZrO_2_ increases the radiopacity of BD, allowing its radiography detection without altering its physicochemical and biological properties.

## Introduction

Mineral trioxide aggregate (MTA) is an endodontic repair material used for root perforation, root-end filling and vital pulp therapy, due to its sealing capability, biocompatibility and ability to induce mineralization^1^ ^-^ ^3^. It is composed of Portland cement clinker (80%) and bismuth oxide (20%) as a radiopacifier^4^. However, MTA is difficult to manipulate and insert into cavities, has a long setting time^5^ and causes tooth discoloration due to the chemical reaction of Bi_2_O_3_ with the dentin matrix^6^.

In an attempt to improve the drawbacks of the MTA, tricalcium silicate-based cements have been developed. One such formulation is Biodentine^®^ (BD) (Septodont; Saint-Maur-des-Fossés, France) which was developed for use as a bioactive dentin substitute, and has been indicated for coronal and radicular restorations, pulp capping, pulpotomy, root repair and root-end filling^7^ ^-^ ^9^. BD powder contains 80% tricalcium silicate, 15% calcium carbonate and 5% zirconium oxide (ZrO_2_), which is used as a radiopacifier agent. The mixing liquid is composed of water, calcium chloride and a hydrosoluble polymer^7^. Studies have shown that this cement has better handling conditions^10^ and lower setting time in comparison with MTA^11^. Moreover, BD has biocompatibility, bioactivity and ability to induce mineralized tissue formation^9^ ^,^ ^12^.

Despite of the good properties of BD, researchers who have used it as root-end filling have reported that its primary clinical limitation is low radiopacity, which hinders radiographic assessment of treatment and follow-up^8^ ^,^ ^13^. Considering the appropriate properties of tricalcium silicate-based cements associated with the radiopacifiers ZrO_2_ or calcium tungstate (CaWO_4_)^14^ ^-^ ^17^, an option to improve the radiopacity of BD is to associate it with these radiopacifiers.

On the other hand, there is no consensus among *in vitro* studies that evaluated the radiopacity of BD. Some studies showed radiopacity lower^18^ ^,^ ^19^ and others higher^16^ ^,^ ^20^ than that recommended by the International Standards Organization^21^, which is at least 3 mm Al.

ISO 6876:2012^21^ recommends that radiopacity of endodontic materials must be evaluated in conventional radiographic films. However, nowadays, the radiopacity of these materials has been evaluated using digital images^20^ ^,^ ^22^. Nonetheless, there is no consensus on how digital radiography influences the radiopacity of materials. Rasimick, et al.^23^ (2007) reported that barium-containing materials tend to be 13% more radiopaque in radiographs obtained by digital sensor than on conventional film. On the other hand, other endodontic materials appeared to be from 7% to 20% less radiopaque on radiographs obtained by photostimulable phosphor plates than on the conventional type^24^. However, the only studies that showed radiopacity of BD higher than 3 mm Al used photostimulable phosphor plates^16^ ^,^ ^20^. Therefore, it is important to evaluate the radiopacity of BD and BD associated with CaWO_4_ or ZrO_2_, using conventional and digital radiography systems, and, in addition, to evaluate the effects on the physicochemical and biological properties of BD when these radiopacifiers are added.

The aim of this study was to evaluate (1) the radiopacity of BD and BD associated with CaWO_4_ or ZrO_2_ using conventional and digital radiography systems, and (2) the physicochemical properties of setting time, pH and solubility, and biological properties of cytocompatibility and osteogenic potential of these cements. The null hypothesis was that there would be no difference in the radiopacity values of BD using conventional or digital radiography systems, and that CaWO_4_ or ZrO_2_ associated with BD would not change its radiopacity nor its biological and physicochemical properties.

## Material and methods

**Figure 1:**
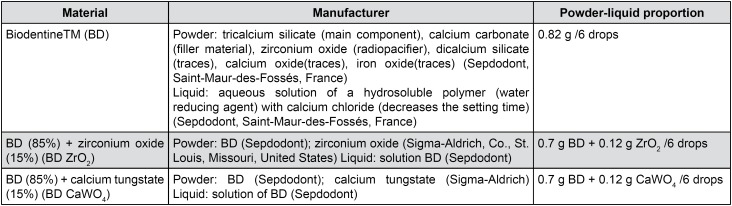
Materials, composition, manufacturer and proportion used

The materials evaluated were BD and BD associated with radiopacifiers ZrO_2_ or CaWO_4_, in a proportion of 85% BD and 15% ZrO_2_ (BD ZrO_2_) or 15% CaWO_4_ (BD CaWO_4_) by weight. The composition, manufacturer, and proportion used for the materials are shown in Figure 1. The cements were mixed according to the instructions of the BD manufacturer.

### Physicochemical properties

#### Radiopacity

Eight specimens measuring 10 mm in diameter by 1 mm wide were made for each material, according to the ISO 6876:2012^21^ specification. The specimens were stored at 37°C and 95% humidity for 24 hours and, subsequently, they were radiographed using conventional or digital radiography systems.

– Conventional radiography: The specimens were placed on occlusal radiographic E-speed films (Kodak; Rochester, NY, USA) along with an 8-step aluminum step-wedge with 2 mm incremental steps for radiographic exposure. The standard geometric configuration was fixed at a distance of 320 mm source-to-object and vertical and horizontal angulations of zero degrees of the X-ray beam. An X-ray unit Focus 50540 (Instrumentarium Dental; Tuusula, Finland), operating at 65 kVp and 7 mA using an exposure time of 0.25 seconds was used^22^. The radiographic films were digitized with a scanner^22^ or with a digital photographic camera^25^. A scanner ScanMaker i800 (Microtek; Hsinchu City, Taiwan) with 300 DPI resolution, and Microtek Scan Wizard 5 (Microtek) software were used. The digital photographic camera EOS T1 (Canon; Tokyo, Japan) with macro lens of 100 mm was used with the following parameters: lens-to-object distance of 58 cm, ISO 200, aperture of 6.3 shutter and speed of 1/40 s.
– Digital radiography: Each specimen along with the aluminum step-wedge were placed on the digital sensors CMOS Fona CDR Elite (FONA Dental; Bratislava, Eslovaquia), CMOS Kodak (rvg 6100, Kodak Co.; Rochester, NY, USA) or on the photostimulable phosphor plate Digora (Soredex; Tuusula, Finland) for radiographic exposure. The standard geometric configuration of the X-ray beam was the same as the one used to obtain conventional radiographs. An X-ray unit (Instrumentarium Dental) was used, operating at 65 kVp and 7 mA and using an exposure time of 0.16 seconds^22^.

The images obtained through all radiographic systems were evaluated using the software Photoshop CC 2015 for Windows (Adobe Systems Incorporated; Mountain View, CA, USA), by measuring the grayscale to determine the equivalence of radiopacity of the cements in millimeters of aluminum (mm Al), using the mathematical formula by Húngaro-Duarte, et al.^25^ (2009).

#### pH analysis

Ten polyethylene tubes measuring 10 mm long and 1 mm in diameter were filled with each material (n=10). Thereafter, each specimen was immersed individually in a plastic flask with a lid, containing 10 mL of deionized water, then stored at 37°C, throughout the experimental time intervals of 1, 3, 10, 20 and 30 days. At each time interval, the pH of the solution was measured with a previously calibrated digital pH meter (Digimed; São Paulo, SP, Brazil). As control, the pH of deionized water without immersed material was measured.

#### Setting time

The cements were inserted into ring-shaped metal molds measuring 10 mm in diameter and 1 mm wide (n=6), kept at 37°C and 95% humidity. To determine the setting time, a Gillmore needle with mass of 100 g ±0.5 and diameter of 2.0 mm ±0.1 was used according to the ISO 6876:2012^21^ specification. The setting time of the cements was considered the time when the needle failed to leave marks on the surface of the specimens.

#### Solubility

The solubility assay was performed according the methodology by Carvalho-Junior, et al.^26^ (2007). The cements were manipulated and placed in a silicone mold of 7.75 mm in diameter and 1.5 mm wide (n=5). A 5-cm nylon wire was placed in the center of the specimens before the material was put into the mold. The specimens were stored at 37°C and 95% humidity for 3 times the length of their setting time. Afterwards, the specimens were removed from the mold, weighed on a 0.001 g precision scale (A & D Engineering; Bradford, MA, USA), and suspended from the lid by means of nylon wires, inside plastic flasks containing 7.5 mL of deionized water. The flasks were stored at 37°C for seven days, then the specimens were removed, rinsed and placed in a silica dehumidifier. The mass was measured every 24 hours after the experiment, until the mass stabilized. The material solubility was expressed as a percentage of the original mass.

The power of the statistical tests was 99% for the results of pH, solubility and setting time, and 87% for radiopacity, with α=0.05.

### Cytocompatibility and osteogenic potential

#### Cell culture and preparation of cement extracts

Human osteoblastic-like cells (Saos-2) were used in this study^2^ ^,^ ^27^. The cells were grown in flaks containing Dulbecco's modified eagle medium − DMEM (Sigma-Aldrich; St. Louis, Missouri, USA), supplemented with 10% fetal bovine serum − FBS (Gibco; Grand Island, NY, USA), penicillin (100 IU/mL) and streptomycin (100 μg/mL) in an atmosphere consisting of 5% CO_2_, with 95% humidity at 37°C.

Each material (0.7 g) was placed in an empty well of a 12-well culture plate (area of 314.0 mm^2^ and 3.0 mm of height) and hydrated with humidified gauze. The plate was kept at 37°C and 95% humidity for 48 hours. After this time, the cements were exposed to ultraviolet light (UV) under laminar flow for 30 minutes to prevent contamination. Five mL of serum-free DMEM were added in each well of the plate in which the material was accommodated. For 24 h, the plate was maintained at 37°C, 95% humidity and 5% CO_2_ to create the extract of each cement^28^. DMEM was used as negative control and 20% dimethyl sulfoxide (DMSO) as positive control.

#### Cell viability assays

Cell viability was evaluated through methyl-thiazol-tetrazolium (MTT) and neutral red (NR) assays. Saos-2 cells were seeded at a density of 1×10^5^ cells/mL in a 96-well plate containing DMEM with FBS 10% for 24 hours. After, the cells were exposed to the cement extracts at 1:1, 1:2, 1:4, 1:8 and 1:12 dilutions (v:v) in serum-free DMEM for 24 hours^2^ ^,^ ^29^. Additionally, the viability of cells exposed to the cement extracts at 1: 8 dilution for 1, 3 and 7 days was assessed by MTT, renewing the extracts every two days.

MTT assay was performed by replacing the cement extracts with 100 µL of a 5 mg/mL MTT solution (Sigma-Aldrich) and the cells were incubated at 37°C, 95% humidity and 5% CO_2_ for 3 h. After this period, the well content was removed and 100 µL of acidified isopropyl alcohol (HCl 0.04 N and isopropyl alcohol) was added to the extract. The absorbance of the solutions was measured in a spectrophotometer at wavelength of 570 nm.

NR assay was performed by replacing the cement extracts with 100 µL DMEM containing 50 µg NR/mL of solution (Sigma-Aldrich). The cells were incubated at 37°C, 95% humidity and 5% CO_2_ for 3 h, and the well content was removed to proceed with solubilization of the colorimetric product in 100 µL of an ethanol solution mixture (50% ethanol and 1% acetic acid). The absorbance of the solutions was measured in a spectrophotometer at wavelength of 570 nm. In both assays, the absorbance readings were normalized with cells exposed to the culture medium. Three independent experiments were performed for both assays.

#### Alkaline Phosphatase (ALP) Activity

ALP activity was assessed by using an ALP kit (Labtest; Lagoa Santa, MG, Brazil). Saos-2 cells (1×10^5^ cells/mL) were seeded in a 96-well plate and exposed to cement extract at 1:8 dilution for 1, 3 and 7 days, renewing the extracts every two days. The absorbance of the solutions was measured in a spectrophotometer at wavelength of 590 nm. Data were expressed as ALP activity normalized with the number of viable cells detected in the MTT assay in the respective culture period, as recommended by Westgard, et al.^30^ (1981).

### Alizarin Red Staining (ARS)

The Saos-2 cells (1×10^4^ cells/mL) were cultivated in a 12-well plate using DMEM supplemented with 50 μg/mL of L-ascorbic acid (Sigma-Aldrich) and 10 mM β glycerophosphate (Sigma-Aldrich). The cells were exposed to the cement extracts at 1:8 dilution for 21 days. The cement extracts, obtained using DMEM supplemented with L-ascorbic acid and β glycerophosphate, were renewed every two days. Immediately afterwards, cells were rinsed with PBS, fixed with 10% paraformaldehyde (Sigma) and stained with 2% ARS (Sigma-Aldrich) with a 4.1 pH. Then, 1 mL of 10% cetylpyridinium chloride solution (Sigma-Aldrich) was added to each well and the plate was incubated for 15 minutes, under shaking. Aliquots of 100 μL of the resuspension of each well were transferred to a 96-well plate and the optical density was measured at 562 nm. Three independent experiments were performed.

### Statistical analysis

The results were analyzed using one-way analysis of variance (ANOVA), or repeated-measures one-way ANOVA, and the Tukey post-test, or two-way ANOVA and the Bonferroni post-test (α=0.05), by using the statistical software GraphPad Prism (GraphPad Software Inc.; San Diego, CA, USA).

## Results

### Physicochemical properties

#### Radiopacity

**Figure 2:**
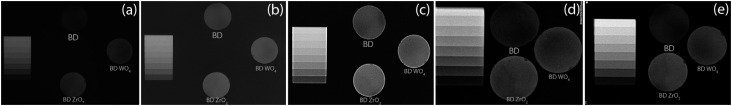
Radiography images obtained with different systems showing the specimens of the materials alongside the aluminum step wedge. (a) occlusal film digitized with a photographic camera; (b) occlusal film digitized with a radiograph scan; (c) photostimulable phosphor plate; (d) Kodak digital sensor; and (e) Fona digital sensor. BD=Biodentine; BD ZrO_2_=Biodentine associated with 15% Zinc Oxide; BD WO_4_=Biodentine associated with 15% calcium tungstate

According to Table 1, in all the digital and convectional radiography systems used, the BD radiopacity did not reach 3 mm Al, as specified by ISO 6876:2012^21^. BD associated with radiopacifiers ZrO_2_ or CaWO_4_ had radiopacity higher than 3 mm Al shown in all radiographic systems. The radiopacity of the materials obtained with the use of a Kodak digital sensor was higher than the values obtained through the other systems (p<0.05). The cements associated to the radiopacifiers showed statistically higher radiopacity than the BD (p<0.05). There was no statistically significant difference between BD ZrO_2_ and BD CaWO_4_ (p>0.05), except for the occlusal film scanned, in which BD CaWO_4_ had higher radiopacity than BD ZrO_2_ (p<0.05). Radiography images of the materials obtained with different systems are shown in Figure 2.

**Table 1:** Mean and standard deviation of the radiopacity values of the materials (mm Al) evaluated by digital or convectional radiography systems

	BD	BD ZrO_2_	BD CaWO_4_
Kodak digital sensor	2.52 (0.11)^a,A^	4.20 (0.30)^a,B^	4.27 (0.39)^a,B^
Fona digital sensor	2.18 (0.03)^b,A^	3.81 (0.14)^b,B^	3.53 (0.37)^c,B^
Photostimulable phosphor plate	2.24 (0.24)^b,A^	3.70 (0.08)^b,c,B^	3.56 (0.37)^c,B^
Occlusal film scanned	2.19 (0.02)^b,A^	3.59 (0.22)^c,B^	3.88 (0.24)^b,C^
Occlusal film photographed	2.06 (0.06)^b,A^	3.71 (0.21)^b,c,B^	3.59 (0.26)^c,B^

Different letters in the columns indicate statistically significant differences between radiographic systems (P<0.05)

#### pH

According to Table 2, the deionized water containing the materials had alkaline pH in all time intervals. No differences were found between groups (p>0.05), except for the control one (deionized water), which had significantly lower pH values than the other groups in all time intervals (p<0.05).

**Table 2:** Mean and standard deviation of pH values of the materials and control in the evaluated time intervals

	BD	BD ZrO_2_	BD CaWO_4_	Control
1 day	11.21 (0.47)^a^	11.28 (0.34)^a^	11.21 (0.35)^a^	6.53 (0.14)^b^
3 days	9.91 (0.72)^a^	9.63 (1.08)^a^	9.48 (0.97)^a^	6.69 (0.19)^b^
10 days	9.53 (1.44)^a^	9.42 (1.32)^a^	9.48 (1.24)^a^	6.69 (0.27)^b^
20 days	9.83 (0.98)^a^	9.06 (1.05)^a^	9.71 (0.95)^a^	6.92 (0.43)^b^
30 days	8.92 (1.37)^a^	8.95 (1.02)^a^	9.75 (1.11)^a^	6.72 (0.18)^b^

Different letters in the lines indicate statistically significant differences between materials (P<0.05)

#### Solubility and setting time

According to Table 3, there was no significant difference between BD and BD ZrO_2_ (p>0.05), both displayed a mass loss lower than 3% and showed no sign of disintegration. BD CaWO_4_ showed higher mass loss (3.63%) in comparison with the other materials (p<0.05), and no sign of disintegration. BD had lower setting time than the other materials (p<0.05). The addition of CaWO_4_ and ZrO_2_ to the BD increased the setting time by 2.5 and 6 minutes, respectively (p<0.05).

**Table 3:** Mean and standard deviation of solubility (% of mass lost) and initial setting time (in minutes) of the materials

	Solubility	Initial setting time
BD	2.28 (0.26)^a^	27.50 (0.57)^a^
BD ZrO_2_	2.27 (0.22)^a^	33.50 (1.73)^b^
BD CaWO_4_	3.63 (0.67)^b^	30.00 (0.81)^c^

Different letters in the columns indicate statistically significant differences between materials (P<0.05)

### Cytocompatibility and osteogenic potential

#### Cell viability assays

**Figure 3:**
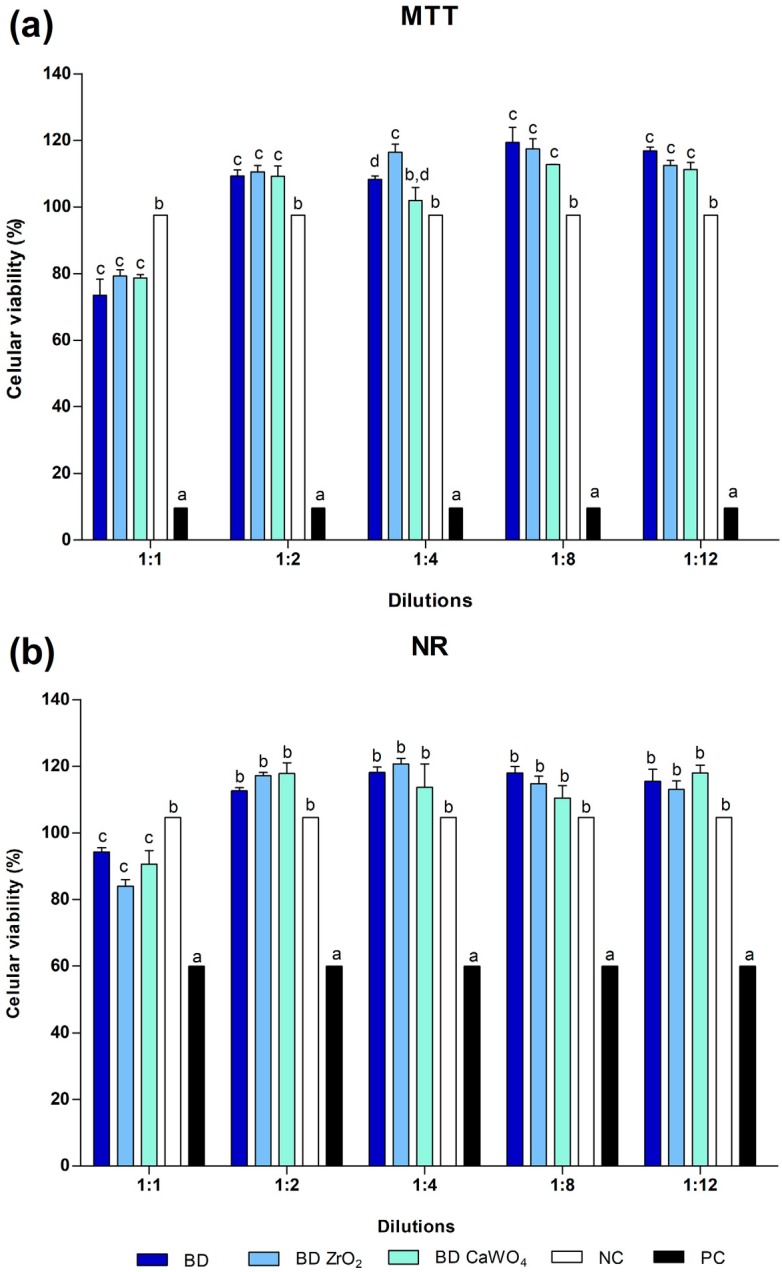
Saos-2 cell viability evaluated by (a) methyl-thiazol-tetrazolium (MTT) and (b) neutral red (NR) assays, after 24 hours of exposure to BD, BD ZrO_2_ and BD CaOW_4_ and controls. At 1:1 dilution, all material groups had lower cell viability than culture medium (negative control). At 1:2, 1:4, 1:8 and 1:12 dilutions, the cement cytocompatibility was greater than or similar to the negative control. Bars with different letters represent significant difference between groups in each dilution. BD=Biodentine; BD ZrO_2_=BD with addition of 15% zirconium oxide; BD CaWO_4_=BD with addition of 15% calcium tungstate; NC=negative control; PC=positive control

MTT and NR assays revealed that, at 1:1 dilution, all material groups had lower cell viability values when compared with culture medium - negative control (p>0.05). At 1:2, 1:4, 1:8 and 1:12 dilutions, the cytocompatibility of the materials was greater than (p<0.05) or similar (p>0.05) to the negative control. In all dilutions, there was no significant difference between BD, BD CaWO_4_ and BD ZrO_2_ (p>0.05), except for the 1: 4 dilution, in which BD ZrO_2_ showed the highest cytocompatibility in the MTT test. In the positive control group (DMSO) there was low cell viability (Figure 3a and 3b). Considering the MTT results, the 1:8 dilution was chosen for ALP activity and ARS assays.

#### ALP activity

**Figure 4:**
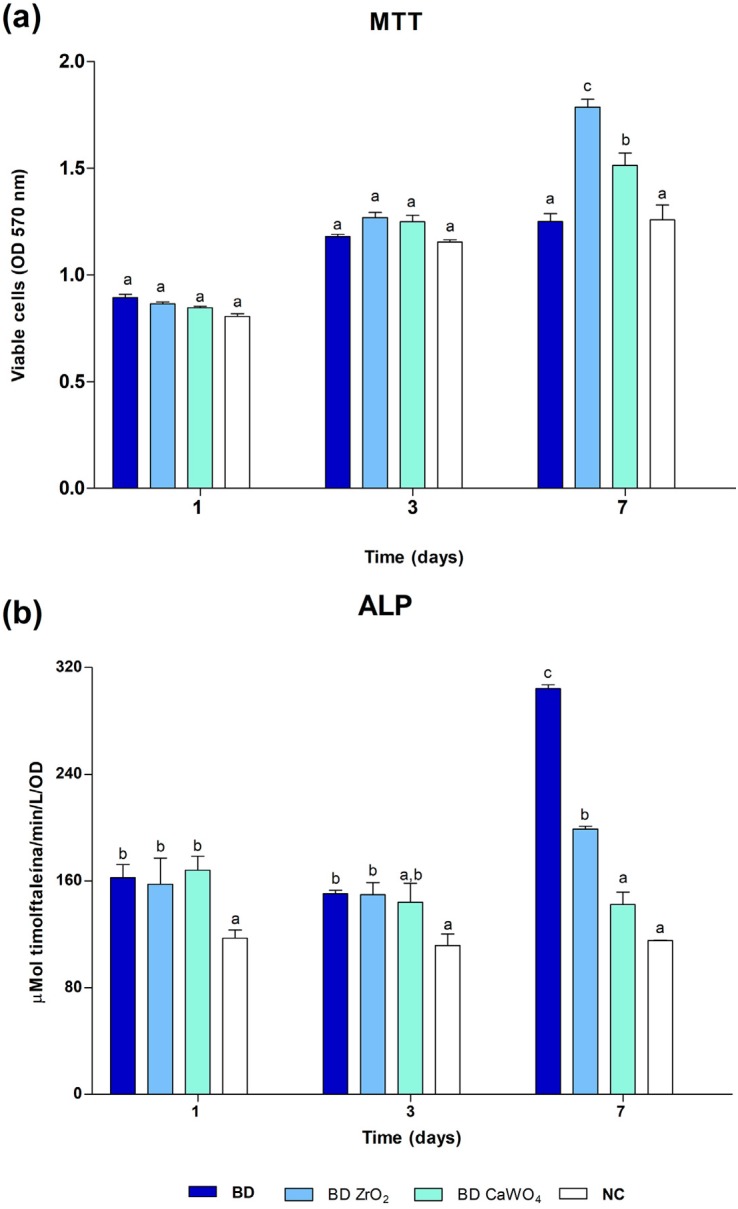
Saos-2 cell viability evaluated by methyl-thiazol-tetrazolium (MTT) assay (a) and alkaline phosphatase (ALP) activity (b) evaluated after exposure to BD, BD ZrO_2_ and BD CaOW_4_ at 1:8 dilution and culture medium (negative control) for 1, 3 and 7 days. The ALP activity of cement groups at 1, 3 and 7 days was similar to or greater than the control group. At 7 days, the highest ALP activity was detected for Group BD followed by Groups BD ZrO_2_ and BD CaWO_4_. There was no significant difference between Group BD CaWO_4_ and the control group. Bars with different letters represent significant differences between groups in each period. BD=Biodentine; BD ZrO_2_=BD with addition of 15% zirconium oxide; BD CaWO_4_=BD with addition of 15% calcium tungstate; NC=negative control

According to Figure 4a, Saos-2 cells exposed to cement extracts had viability similar to (p>0.05) or greater (p<0.05) than the control group (culture medium) at 1, 3 and 7 days. The lowest cell viability was detected on the first day of cell exposure to the cement extracts, increasing over the time intervals of 3 and 7 days. At 7 days, Groups BD ZrO_2_ and BD CaWO_4_ showed higher cell viability values than BD and the control group (p<0.05), however, there was no significant difference between BD and the control group (p>0.05). The ALP activity (Figure 4b) of cement groups at 1, 3 and 7 days was similar to (p>0.05) or greater (p<0.05) than that of the control group. At 7 days, the highest ALP activity was detected for BD, followed by BD ZrO_2_ (p<0.05) and BD CaWO_4_ (p<0.05). There was no significant difference between Group BD CaWO_4_ and the control group (p>0.05).

#### Alizarin red staining

**Figure 5:**
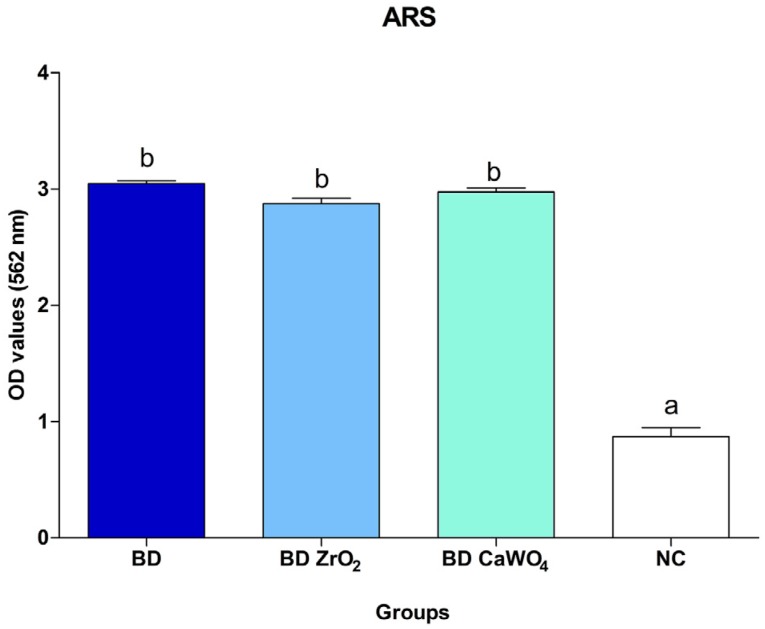
Alizarin red staining (ARS) assay. Comparison of mineralized nodule production after 21 days of cell exposure to BD, BD ZrO_2_ and BD CaOW_4_ extracts and negative control group. All cements induced a greater mineralized nodule production when compared with the negative control group. Bars with different letters represent significant differences between groups. BD=Biodentine; BD ZrO_2_=BD with addition of 15% zirconium oxide; BD CaWO_4_=BD with addition of 15% calcium tungstate; NC=negative control

As observed in Figure 5, all materials induced a greater production of mineralized nodules when compared to the negative control group (p<0.05) after 21 days of cell exposure to the cement extracts. There was no significant difference among the cement groups (p>0.05).

## Discussion

The first aim of this study was to evaluate the radiopacity of BD and BD associated with 15% CaWO_4_ or ZrO_2_. For the manipulation of BD and BD associated with radiopacifiers, 6 drops of liquid were used, instead of 5 as indicated by the manufacturer, because 0.12 grams of radiopacifier were added to the 0.7 grams contained in a capsule of BD. To standardize the quantity of powder contained in each capsule, a total of 0.82 grams was used for BD.

Radiopacity was evaluated using conventional radiography and different digital radiography systems, because, according to literature, the radiopacity of materials may vary between 7% and 20%, depending on the radiography systems used^24^. In this study, BD had lower radiopacity than the 3 mm Al specified by the ISO 6876:2012^21^, in all radiography systems used (2.08 mm Al – 2.52 mm Al). This meant that 5% of ZrO_2_ present in BD^7^ was not sufficient to provide an adequate radiopacity. The radiopacity of BD agreed with the values showed in previous studies, which showed radiopacity of 2.79 mm Al when using digitized conventional radiography^19^, and 2.80 mm Al when using photostimulable phosphor plates^18^. Conversely, studies using photostimulable phosphor plates showed radiopacity of BD around 4 mm Al^16^ ^,^ ^20^. The differences could be due to various factors, such as the X-ray machine, exposure time, tube voltage and source-to-object distance^19^.

BD ZrO_2_ and BD CaWO_4_ had radiopacity between 3.52 and 4.26 mm Al, showing that the addition of the radiopacifiers in the proportion of 15%, resulting in approximately 20% in weight, was sufficient to increase the radiopacity of BD to values higher than the minimum specified by the ISO 6876:2012^21^. The amount of radiopacifier added to BD was based on studies that showed that tricalcium silicate associated with 20% ZrO_2_ or Portland cement with 20% ZrO_2_ or 20% CaWO_4_ exhibited radiopacity higher than 3 mm Al, in addition to adequate physicochemical properties and cytocompatibility^14^ ^-^ ^16^.

The second aim of this study was to evaluate the physicochemical properties setting time, pH and solubility; biological properties of cytocompatibility, and potential for induction of mineralization of BD and BD CaWO_4_ or BD ZrO_2_.

An alkaline medium contributes to osteogenic potential and antibacterial activity of a material^31^. The addition of radiopacifiers did not change the pH of BD CaWO_4_ or BD ZrO_2_ when compared with BD; all cements had alkaline pH in all time intervals. Alkaline pH of BD has been studied in literature^12^ ^,^ ^19^ and it results from the hydration reaction of tricalcium silicate, which forms calcium hydroxide that dissociates, thereby alkalinizing the medium^16^. It is important to inform that BD associated with radiopacifiers showed a better consistency and greater ease of handling in comparison with BD.

Materials that show high solubility may provide inadequate sealing and the presence of gaps in the filling^32^. The ISO 6876:2012^21^ establishes that solubility cannot be greater than 3% of the total mass after 24 hours of immersion of the specimens in water. In this study, BD showed mass loss of 2.28 % after being immersed in water for 7 days. This result was in line with findings of a previous study, which showed BD mass loss of 2.74%, 2.74% and 2.90% at 24 hours, 3 and 10 days of immersion in water, respectively^33^. Opposite to our results, some researchers reported solubility of BD higher than 3% evaluated in periods from 24 hours to 7 days of immersion in water^11^ ^,^ ^34^. The addition of ZrO_2_ did not change the solubility of BD, and the addition of CaWO_4_ increased it to 3.63%. Studies have shown that solubility of tricalcium silicate-based cement was not altered by the addition of ZrO_2_ or CaWO_4_ ^15^ ^,^ ^35^.

The setting time of root canal sealers should be long enough to allow their manipulation and placement in the root canal system^36^. On the other hand, cements with long setting time were more susceptible to dissolution^37^. The initial setting time of BD was 27.5 minutes. Previous studies have shown initial setting times of 16 minutes^19^ or 85.66 minutes^11^ for BD. The initial setting times of BD CaWO_4_ and BD ZrO_2_ were 30 minutes and 33.5 minutes respectively, which represented an increase in setting time of 2.5 minutes for BD CaWO_4,_ and 6 minutes for BD ZrO_2_ in comparison with BD.

Considering the relevance of osteoblast response to mineralized tissue repair, human osteoblastic-like cells (Saos-2) were used in this study^2^ ^,^ ^27^. Simultaneous evaluation of different cell parameters is necessary to provide reliable information on the cytotoxicity of materials. The MTT test for assessing cell metabolic activity is based on succinate dehydrogenase mitochondrial enzyme activity, which converts the tetrazolium salt into formazan crystals that are violet colored. The absorbance of solubilized formazan crystals is proportional to the number of living cells. NR is a cell viability assay based on the incorporation of NR dye into lysosomes/endosomes and vacuoles of living cells. Thus, the loss of NR uptake corresponds to the loss of cell viability^28^.

According to the MTT and NR results of this study, all cements evaluated were cytocompatible. The cytocompatibility of BD has been shown in human dental pulp cells^3^ and osteoblasts-like cells^27^. The addition of the radiopacifiers ZrO_2_ and CaWO_4_ did not prejudice the cytocompatibility of the cement. Studies have shown that tricalcium silicate-based cements associated with these radiopacifiers were not cytotoxic to periodontal and osteoblast-like cells, induced fibroblast proliferation and accelerated the regression of the inflammatory reaction when compared with MTA in subcutaneous rat tissue^14^ ^,^ ^17^.

ALP activity and ARS assays were performed to evaluate the osteogenic potential of the cements. ALP plays a critical role in mineralization^38^. After 7 days of Saos-2 cell exposure to the cement extracts, ALP activity increased, especially for BD and BD ZrO_2_, when compared with untreated cells. These results were in line with those of a previous study that showed that BD and tricalcium silicate-based cement associated with 30% ZrO_2_ showed potential to induce mineralization^27^. ARS is a test used to evaluate calcium deposits in cell culture. All materials induced greater production of mineralized nodules when compared with the control group. These results agreed with studies which revealed that BD induced similar or greater production of mineralized nodules than unexposed cells^27^. In summary, BD, BD associated with ZrO_2_ or CaWO_4_ had cytocompatibility, induced ALP activity and production of mineralized nodules necessary to promote endodontic repair.

## Conclusions

BD radiopacity was lower than 3 mm Al, as shown by the conventional and digital radiography systems, and adding 15% ZrO_2_ or CaWO4 was sufficient to increase it to values higher than the minimum specified by ISO 6876:2012^21^ (>3 mm Al). BD associated with radiopacifiers showed suitable properties of setting time, pH and solubility, except for BD CaWO_4_, which exhibited higher solubility than BD and BD ZrO_2_. All cements evaluated had cytocompatibility and potential to induce mineralization in Saos-2 cells. The results showed that the addition of 15% ZrO_2_ increases the radiopacity of BD allowing its radiography detection, without altering its physicochemical and biological properties.
